# Effects of Beetroot Juice Supplementation on Performance and Fatigue in a 30-s All-Out Sprint Exercise: A Randomized, Double-Blind Cross-Over Study

**DOI:** 10.3390/nu10091222

**Published:** 2018-09-04

**Authors:** Eduardo Cuenca, Pablo Jodra, Alberto Pérez-López, Liliana G. González-Rodríguez, Sandro Fernandes da Silva, Pablo Veiga-Herreros, Raúl Domínguez

**Affiliations:** 1 GRI-AFIRS, Escuela de Ciencias de la Salud, TecnoCampus-Universidad Pompeu Fabra, Mataró, 08005 Barcelona, Spain; educuen@hotmail.com; 2 Faculty of Health Sciences, University Alfonso X El Sabio, 28691 Madrid, Spain; pjodrjim@uax.es (P.J.); liligoro@uax.es (L.G.G.-R.); pveigher@uax.es (P.V.-H.); 3 Department of Education Sciences, University of Alcalá, 28805 Alcalá de Henares, Spain; 4 Department of Biomedical Sciences, Faculty of Medicine and Health Sciences, University of Alcalá, 28805 Alcalá de Henares, Spain; alberto_perez-lopez@hotmail.com; 5 Investigation Group Valornut, Department of Nutrition, Faculty of Pharmacy, University Complutense de Madrid, 28691 Madrid, Spain; 6 Studies Research Group in Neuromuscular Responses (GEPE N), University of Lavras, 37200-000 Lavras, Brazil; sandrofs@gmail.com; 7 Faculty of Health Sciences, University Isabel I, 09004 Burgos, Spain

**Keywords:** nitric oxide, nitrates, muscle power, muscle fatigue

## Abstract

As a nitric oxide precursor, beetroot juice (BJ) is known to enhance high-intensity exercise performance (80–100% VO_2max_) yet its impacts on higher intensity sprint exercise (>100% VO_2max_) remain to be established. This study sought to examine the effects of BJ supplementation on performance and subsequent fatigue during an all-out sprint exercise. Using a randomized cross-over, double-blind, placebo-controlled design, 15 healthy resistance-trained men (22.4 ± 1.6 years) ingested 70 mL of either BJ or placebo. Three hours later, participants undertook a 30-s all-out Wingate test. Before and after the sprint exercise and at 30 s and 180 s post-exercise, three countermovement jumps (CMJ) were performed and blood lactate samples were obtained. Compared to placebo, BJ consumption improved peak (placebo vs. BJ, 848 ± 134 vs. 881 ± 135 W; *p* = 0.049) and mean (641 ± 91 vs. 666 ± 100 W; *p* = 0.023) power output and also reduced the time taken to reach W_peak_ in the Wingate test (8.9 ± 1.4 vs. 7.3 ± 0.9 s; *p* = 0.003). No differences were detected in the fatigue index. In addition, while over time CMJ height and power diminished (ANOVA *p* < 0.001) and blood lactate levels increased (ANOVA *p* < 0.001), no supplementation effect was observed. Our findings indicate that while BJ supplementation improved performance at the 30-s cycling sprint, this improvement was not accompanied by differences in fatigue during or after this type of exercise.

## 1. Introduction

Dietary nitrate supplementation has been described as a potential ergogenic aid for high-intensity exercise efforts (80–100% VO_2max_) as it reduces the oxygen cost of ATP synthesis and ATP cost of muscle contraction thus improving muscle contraction/relaxation, force and power production [1,2,3]. However, the impacts of nitrate supplementation on all-out sprint exercise performance (>100% VO_2max_), and particularly its effects on the fatigue induced by this mode of exercise [4,5,6] have been scarcely addressed.

Ingested nitrate (NO_3_^−^) is a well-known precursor of nitric oxide (NO) in humans [7]. Around 25% of circulating NO_3_^−^ is taken up by salivary gland acinar cells in a process facilitated by sialin [8,9]. Oral microorganisms, particularly those on the posterior aspect of the tongue, initiate the reduction of NO_3_^−^ into nitrite (NO_2_^−^), which subsequently in the stomach and gut, can be converted into NO and be absorbed under hypoxic conditions [8,9,10]. The majority of the remaining NO_3_^−^ and NO_2_^−^ molecules that reach the intestine are absorbed by this organ increasing NO levels in blood [9]. NO offers several exercise adaptation benefits [11] through its effects of inducing vasodilatation, reducing blood viscosity, and promoting muscular oxygen perfusion and gas exchange [12]. In skeletal muscle, NO reduces oxidative stressor production and promotes mitochondrial biogenesis and efficiency [13,14]. Moreover, NO it is also able to increase force and power production during muscle contraction, decreasing the cost of ATP needed as well as the oxygen required to synthesize ATP [1,2,3].

Beetroot juice (BJ) is a NO_3_^−^-rich supplement commonly used because of its high betacyanin and polyphenol contents that promote NO synthesis to a greater extent than other NO_3_^−^ salts [15,16]. The ergogenic effect of NO_3_^−^ supplementation was initially observed in terms of metabolic adaptations to endurance training [17]. However, despite the known impact of BJ on aerobic performance, recent data indicate a potential effect of NO_3_^-^-rich supplements on anaerobic exercise [4]. 

Interestingly, the observed benefits of BJ only seem to affect type II muscle fibers [11]. In these fibers, NO stimulates calcium release into the sarcoplasm via calsequestrin upregulation [18] and reduces the phosphocreatine degradation rate, decreasing ATP cost across several ranges of exercise intensity [19]. During sprint exercise (>100% VO_2max_), type II muscle fibers are mainly recruited to satisfy the high muscle contraction demands. In these glycolytic fibers, exercise leads to a reduced pH in comparison to oxidative fibers. Intra-cell acidity also promotes the reduction of NO_2_^−^ to NO [8]. In turn, the increase in NO availability may diminish the ATP and phosphocreatine required by each muscle contraction with the consequence of an ergogenic effect of NO_3_^−^ supplementation in sprint exercise achieved by improving power production and attenuating the fatigue induced by this exercise mode [20,21].

However, despite acute BJ administration emerging as an effective strategy to improve different modes of exercise performed to exhaustion [22], the influence of this supplement has been scarcely explored in sprint exercise [1,2,3,20,23,24]. Two studies have shown that BJ supplementation increases peak power output in a 3–4 s [23] or 30 s cycle ergometer exercise [20,23,24]. However, the benefits of BJ on the muscle power produced in a vertical jump have not been investigated. The countermovement jump (CMJ) is a useful test to explore the muscle contractile properties and neuromuscular performance of the lower-limbs [25]. This test has been extensively used in high-intensity sports in which the stretch-shortening cycle plays a pivotal role [26]. Further, given that fatigue can be defined as a reduction in strength or power regardless of the ability to sustain a required task [27], conducting the CMJ before and after an extenuating task is an effective method of monitoring muscle fatigue [28]. In this context, the present study was designed to examine the effects of BJ, as a NO_3_^−^-rich supplement, on performance at a single 30-s all-out sprint exercise and the fatigue caused by the exercise bout. Our working hypothesis was that BJ intake would increase the peak power generated by muscle contraction and reduce the time needed to achieve this peak power output with the consequence of diminished neuromuscular fatigue after the sprint.

## 2. Materials and Methods 

### 2.1. Participants

Fifteen young men (age 22.4 ± 1.6 years, height 178 ± 6 cm, weight 76.9 ± 10.3 kg) were recruited. All subjects had at least 18 months of experience with resistance exercise, training 3 sessions per week (e.g., bench press and leg press 1RM were 1.0 and 1.5-fold higher than their body mass weight, respectively) and were familiar with the 30-s all-out Wingate and CMJ tests. Subjects were instructed to refrain from taking sports supplements, medical supplements or any ergogenic aids during the 3 months before the tests and were excluded if they failed to comply. Further exclusion criteria were smoking or cardiovascular, pulmonary, metabolic or neurologic disease. 

Candidate participants were first informed of the experimental protocol before giving their written consent. The study was approved by the Ethics Committee of Alfonso X University in (code 1.010.704) accordance with the latest version (7th) of the Declaration of Helsinki.

### 2.2. Experimental Design

The study design was randomized cross-over, placebo-controlled and double-blind. Participants reported to the laboratory on two separate days under the same experimental conditions (72 h between sessions, 0.5 h difference in test initiation). Participants were instructed to avoid any form of exercise in the 72 h leading up to each test. 

**Figure 1:**
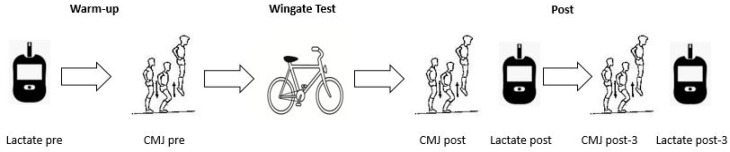
Experimental procedure.

In session 1, participants were subjected to a preliminary assessment of body composition and underwent a familiarization session of the experimental protocol. Then, on two separate occasions (sessions 2 and 3) as they arrived at the laboratory, participants were provided with a supplement containing either placebo (placebo) or BJ. The trial was double-blinded such that one researcher (P.V.-H.) allocated all the participants’ drinks in a counter-balanced fashion (in each trial 50% of participants ingested placebo and 50% ingested BJ beverages) with random assignment to each supplement (using Excel, Microsoft, Washington, DC, USA) and this researcher did not take part in the subsequent experimental procedures or statistical analysis of data. Three hours after taking the supplement, all participants performed a 30 s all-out Wingate test on a Monark ergometer (Ergomedic 828E, Vansbro, Sweden), as previously described [19]. Strong verbal encouragement was provided in all the sprint tests. In addition, data were collected in three CMJ jumps and blood samples for lactate determination were obtained in duplicate before (Pre) and after the sprint exercise at 30 s (Post) and 180 s post-exercise (Post-3). The study procedure is illustrated in Figure 1. 

### 2.3. Placebo vs. BJ Ingestion 

After an overnight fast, participants reported to the laboratory 3 h before the first CMJ jump test. Upon arrival, they were provided with either 70 mL of BJ (containing 6.4 mmol of NO_3_^−^) or the same drink lacking NO_3_^−^ (placebo, 0.04 mmol of NO_3_^−^) (Beet-It-Pro Elite Shot, James White Drinks Ltd., Ipswich, UK) as described elsewhere [20]. 

All participants were instructed to follow a diet sheet the day before each trial that consisted of 60% carbohydrates, 30% fat and 10% proteins. Dietary NO_3_^−^ was limited by providing subjects a list of NO_3_^−^-rich foods (e.g., beetroot, celery or spinach) they should avoid in the 48 h before each trial. Also, in the 24 h leading up to each test, subjects were encouraged to avoid brushing their teeth or use an oral antiseptic rinse, or ingest gum, sweets or stimulants (e.g., caffeine) that could alter the oral microbiota and interfere with NO_3_^−^ reduction. 

### 2.4. Sprint Performance Variables 

Power output (W) was monitored second-by-second in all sprints. Mean power output (W_mean_) was calculated as the average power generated during the 30-s test. Peak power output (W_peak_) was taken as the highest W value recorded. The time (s) taken to reach W_peak_ was also recorded. Minimum power output (W_min_) was considered as the lowest W value recorded during the 10 last seconds of the test. Finally, the fatigue index (FI) was calculated using the equation: FI = (W_peak_ − W_min_)/W_peak_. In addition, mean power output in each Wingate test was calculated for the entire test (30 s) and at 10 s (W_mean0–10s_, W_mean10–20s_ and W_mean20–30s_) and 15 s intervals (W_mean0–15_ and W_mean15–30s_) as described elsewhere [19].

### 2.5. Neuromuscular Fatigue

Neuromuscular fatigue in the legs was measured as the loss of height and power in a CMJ test performed on a force platform (Quattro Jump model 9290AD; Kistler Instruments, Winterthur, Switzerland) [28,29,30]. Participants were highly familiarized with this vertical jump test. Two CMJ were performed before (Pre) and after the Wingate test at 30 s (Post-1) and 180 s post-exercise (Post-3). At each time-point, mean values of height (cm), mean power (CMJ_Wmean_) and peak power (CMJ_Wpeak_) were recorded. 

### 2.6. Blood Lactate

Before the first CMJ and immediately after the subsequent vertical jumps, capillary blood samples (5 µL) were obtained from the index finger of the right-hand for lactate determination using a Lactate ProTM 2 LT-1710 Instrument (Arkray Fatory Inc., KDK Corporation, Shiga, Japan).

### 2.7. Statistical Analysis

The Shapiro-Wilk test was first performed to assess the distribution of the data. Then paired *t*-tests for normally-distributed data and the Wilcoxon test for non-normally distributed variables (Time-to-W_peak_, W_0–15s_, W_15–30s_, W_10–20s_ and W_20–30s_) were used to compare all sprint variables between the experimental conditions (placebo vs. BJ). A two-way ANOVA for repeated measures was also used to compare placebo vs. BJ for two between-subject conditions: supplementation (placebo vs. BJ) and time (pre-exercise, 30 s post-exercise and 180 s post-exercise). Before the ANOVA, we confirmed there was no violation of the sphericity assumption using Mauchly’s test of sphericity. Holm-Bonferroni was used as post-hoc test when significant differences were detected. Values are provided as the mean ± standard deviation (SD). Significance was set at *p* < 0.05. All statistical tests were performed using the software package SPSS v.18.0 (SPSS Inc., Chicago, IL, USA).

## 3. Results

### 3.1. Sprint Performance Variables

**Figure 2:**
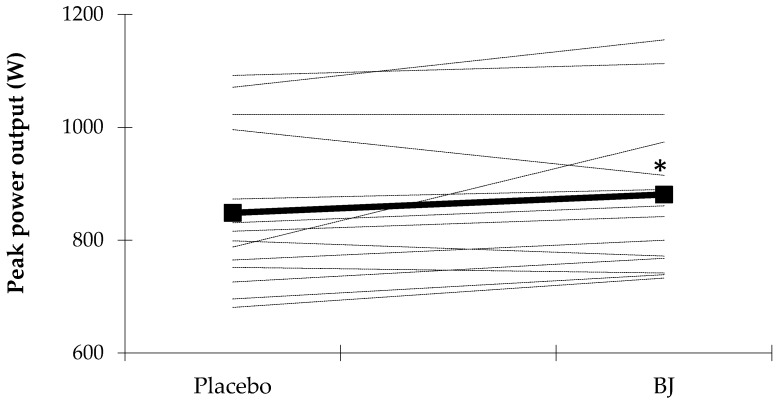
Effects of placebo and BJ intake on W_peak_ after sprint exercise. Means and individual values are shown as a bold or dotted line respectively. * *p* < 0.05 compared to placebo. BJ, beetroot juice.

The effects of placebo and BJ on the 30-s all-out sprint test are shown in Table 1. Compared to placebo, BJ supplementation increased W_peak_ (~3.8%; *p* = 0.049) and W_mean_ (~4.0%; *p* = 0.023), while reduced time to W_peak_ (~18%; *p* = 0.003). In 12 of the 15 participants, W_peak_ was higher after BJ administration compared to the placebo condition (Figure 2). In contrast, no significant differences were observed in W_min_ (~4.4%; *p* = 0.064) or FI (~0.22%; *p* = 0.914).

**Figure 3:**
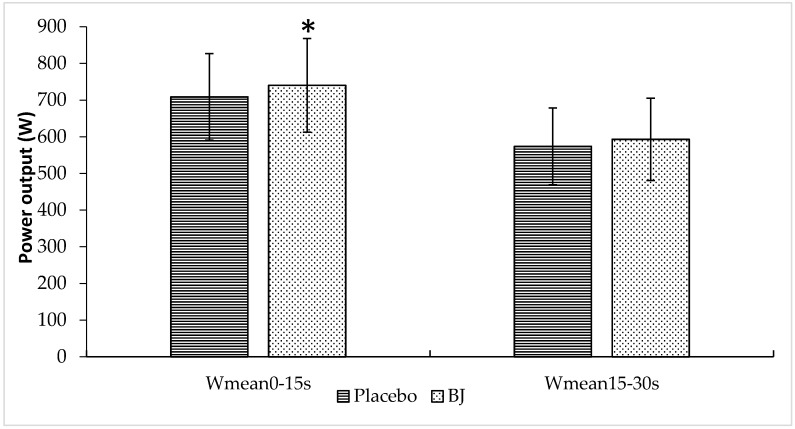
Effects of placebo and BJ intake on W_mean_ values recorded over 15 s intervals (A, W_0–15s_; B, W_15–30s_) after the sprint. * *p* < 0.05 compared to placebo.

Values of W_mean_ were recorded in 10 and 15 s intervals. Figure 3 displays W_mean_ values in 15 s intervals (W_0–15s_ and W_15–30s_). An increased W_mean_ was observed after BJ intake compared to placebo during the first 15 s of the sprint (placebo vs. BJ, 709 ± 113 vs. 740 ± 122 W_0–15s_; *p* = 0.017), while no significant differences were recorded during the last 15 s (placebo vs. BJ, 574 ± 80 vs. 593 ± 87 W_15–30s_; *p* = 0.173).

**Figure 4:**
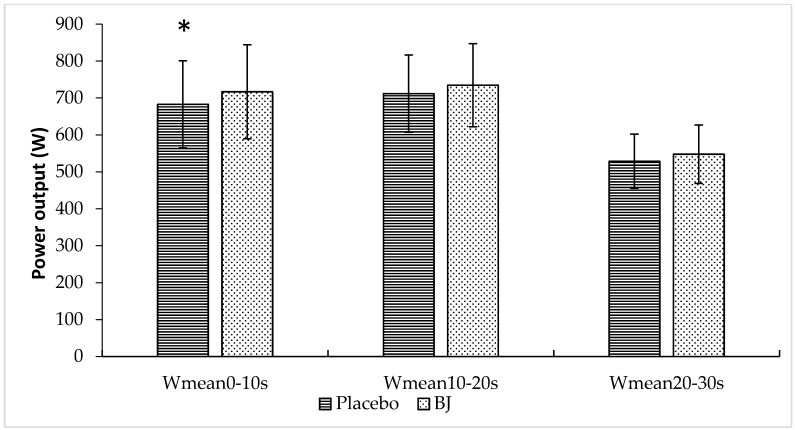
Effects of placebo and BJ intake on W_mean_ values recorded over 10 s intervals (W_mean0–10s_, W_mean10–20s_ and W_mean20–30s_) after the sprint. Values are means ± standard deviation. * *p* < 0.05 compared to placebo.

Figure 4 provides W_mean_ values in 10 s intervals (W_0–10s_, W_10–20s_ and W_20–30s_). Compared to placebo, a significant increase in W_mean_ was observed after BJ intake during the first 10 s interval (placebo vs. BJ, 683 ± 118 vs. 717 ± 127 W_0–10s_; 5.0%, *p* = 0.043), while no significant differences emerged for the intervals 10–20 s (placebo vs. BJ, 712 ± 105 vs. 735 ± 113 W_10_–_20s_; 3.2%, *p* = 0.078) or 20–30 s (placebo vs. BJ, 529 ± 73 vs. 548 ± 79 W_20–30s_; 3.6%, *p* = 0.30).

### 3.2. Neuromuscular Fatigue and Blood Lactate Concentrations

The effects of placebo and BJ intake on neuromuscular fatigue measured through the CMJ test are shown in Table 2. The 30-s all-out Wingate test led to significant reductions in CMJ_height_, CMJ_Wpeak_ and CMJ_Wmean_ (ANOVA time effect, *p* < 0.001). Compared to Pre, a significant decrease was observed at Post and Post-3 in CMJ_height_ (Pre vs. Post, ~38%; Pre vs. Post-3, ~19%; *p* < 0.001), CMJ_Wpeak_ (Pre vs. Post, ~28%; Pre vs. Post-3, ~10%; *p* < 0.001) and CMJ_Wmean_ (Pre vs. Post, ~21%; Pre vs. Post-3, ~14%; *p* < 0.001); while a significant increase was observed for Post-3 compared to Post in all variables (~24% CMJ_height_, ~22% CMJ_Wpeak_, ~21% CMJ_Wmean_; *p* < 0.001). No supplementation or interaction effects (supplement × time) were observed. 

**Figure 5:**
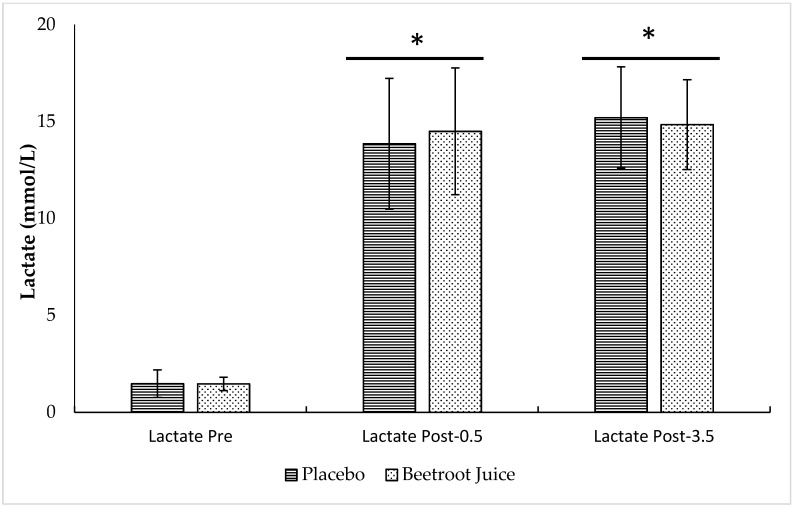
Blood lactate concentrations recorded after the sprint for the placebo and BJ conditions. Values are means ± standard deviation. * *p* < 0.05 compared to Pre. Pre, before sprint exercise; Post-0.5, 0.5 min post-exercise; Post-3.5, 3.5 min post-exercise.

Figure 5 illustrates the blood lactate values recorded after the sprint test. Blood lactate concentration was significantly higher after the 30-s all-out Wingate test (ANOVA time effect, *p* < 0.001). Compared to Pre (placebo, 1.47 ± 0.71 mmol/L; BJ, 1.47 ± 0.35 mmol/L), blood lactate was significantly higher at the time points Post-0.5 (placebo, 13.86 ± 3.37 mmol/L; BJ, 14.49 ± 3.27 mmol/L; *p* < 0.001) and Post-3.5 (placebo, 15.20 ± 2.62 mmol/L; BJ, 14.84 ± 2.32 mmol/L; *p* < 0.001). No supplementation (ANOVA supplementation effect, *p* = 0.858) or interaction effects (ANOVA supplement × time effect, *p* = 0.719) were detected.

## 4. Discussion

The findings of our study indicate that BJ supplementation enhances peak and mean power output, particularly during the first half of a 30-s all-out sprint test, reducing the time taken to reach peak power output. Despite this improved sprint performance, neuromuscular fatigue caused by this exercise mode was similar after the intake of BJ or placebo. These observations suggest that NO_3_^−^-rich supplements enhance sprint performance without producing cumulative impacts on fatigue levels. 

NO_3_^−^ supplementation has been linked to an increase in W_peak_ generated by leg extension in an isokinetic machine at several angular velocities (from 0 to 6.28 rad/s) in healthy subjects (~5–6%) [2,31] and patients with heart disease (~12%) [32]. There are two reports in the literature of investigations examining the effects of an acute dose of BJ on a 30-s all-out Wingate test [19,23]. In the study by Domínguez et al. [20], a significant increase in W_peak_ was observed (~6%) while Rimer et al. [23] observed no such effect. It should be mentioned that in the study by Rimer’s group [23], the 30-s Wingate test was performed after 4 series of 3–4 s all-out sprint trials and 5 min of passive rest; and despite the lack of difference in the Wingate test, the delta change in peak power output produced in the 3–4 sprints indicated an increase of ~6.0% after BJ intake compared to placebo. In the present study, a similar increase in peak power output was observed after the 30-s all-out Wingate test (~4%) and this performance improvement seems to occur during the first 15 s of the sprint and hereafter decline. These data indicate that BJ supplementation may cause a transient ergogenic elevation of peak power output during the first few seconds of sprint exercise, and that this effect could be attenuated after several doses of BJ [6].

The use of an isokinetic or isoinertial cycle ergometer for the sprint test may be a confounding factor when examining the ergogenic effect of BJ supplementation [19,23]. In this study, we used an isoinertial cycle ergometer, which measures power output based on a variable pedaling rate at a fixed load (7.5% body mass) [20]. In contrast, using an isokinetic cycle ergometer, the pedaling rate is predetermined [23]. Pedaling rate is strongly related to the angular velocity of the knee and hip, and can be used as an indicator of muscle contraction velocity [33] and type II motor unit recruitment [34]. An ergogenic effect of BJ intake has been observed not only in sprint exercise [20] but also in other tasks (e.g., leg extension) under elevated angular velocities [2,31,32]. Consistent with this idea, the present data revealed a greater effect of BJ on sprint performance (W_peak_ and time to W_peak_) for the higher angular velocities. 

Animal studies have shown that NO increases acetylcholine activity, particularly in type II motor units, which amplify depolarization of the muscle fibers [35] whereas BJ supplementation induces the elevation of intracellular Ca^2+^ concentrations accompanied by calsequestrin 1 and dihydropyridine receptor upregulation in fast-twitch muscles [18]. Although these mechanisms have not yet been proven in humans, NO_3_^−^ supplementation likely increases force production by inducing type II muscle fiber depolarization and increasing myoplasm Ca^2+^ concentrations facilitating muscle contraction [18,36] by increasing the number of actin-myosin cross-bridges [37]. This improvement in muscle force production in response to BJ consumption has been detected as a higher rate of force development (RFD) [37] through increased peak power output, the time taken to reach that power output and a faster reaction time [4]. In effect, Time to W_peak_ and reaction time are key factors in sports performance, particularly in disciplines in which acceleration determines performance [38,39]. Here, BJ supplementation led to a pronounced reduction in Time to W_peak_ during a 30-s all-out Wingate test, coinciding with previous data in which the increase in W_peak_ was accompanied by a shorter time needed to reach W_peak_ [20]. A reduced Time to W_peak_ was also found when a transient increase in W_peak_ was not detected after prolonged doses of BJ supplements and repeated sprint exercise [6]. In these two previous studies [6,20], the shortened Time to W_peak_ was lower (~0.7 and ~0.2 s, respectively) than the difference observed here (~1.6 s). The greater improvement in Time to W_peak_ reported here may be explained by a reduced level of anaerobic training of our subjects compared to participants of the studies by Dominguez et al. [20] and Jonvik et al. [6], who were well-trained in anaerobic disciplines.

Anaerobic pathways supply ~75% of energy requirements in a 30-s all-out sprint exercise [40,41]. During the first 6 s, ready to use sources of energy are needed to produce maximal peak power output in the shortest time possible. Accordingly, free ATP and PCr stores are critical during the initial part of a sprint [42]. At this time (first 5–10 s), a marked depletion in PCr stores occurs and this compromises power output coinciding with the time at which glycolysis attains its maximum rates [43]. Along with an increased force production capacity, BJ supplementation leads to the reduced ATP cost of muscle contraction [19,44] perhaps by reducing PCr degradation rates. The reduced ATP requirements of muscle contraction together with the maintenance of free ATP and PCr stores promoted by NO_3_^−^ supplementation may give rise to a higher power output during a longer period of time coinciding with the increase in mean power output produced during the first 15 s of the sprint after BJ intake. 

Since BJ consumption led to elevated peak and mean power output during the first 15 s of the sprint, we could argue that the muscular fatigue that takes place during the last 15 s and at the end of the sprint will be exaggerated. 

The fatigue index calculated during the sprint indicated no differences between the supplements. In addition to the mentioned maintenance of anaerobic sources of energy production, the contribution of aerobic energy production increases during the last 15 s of a Wingate test [41,43]. Since NO_3_^−^ supplementation is known to reduce the oxygen cost of ATP synthesis [45] and to preserve ATP and PCr stores [19], the lack of differences between supplements (placebo vs. BJ) may be explained by a higher capacity of NO_3_^−^ to induce ATP store maintenance and thus reduce the cost of its synthesis by both aerobic and anaerobic sources. 

Immediately after the sprint exercise, two CMJ jumps were performed at 30 s and 180 s. CMJ is a vertical jump test that assesses muscle contractile properties and neuromuscular performance (anaerobic power) of the lower-limbs [46,47]. Variables such as CMJ height and power have also been used as indicators of neuromuscular fatigue [48,49]. Some authors have argued that the CMJ test after extenuating exercise [28] serves to assess muscle capacity to replenish ~50% of depleted PCr stores at 30-s post-exercise [50] and to recover almost completely depleted PCr stores at 180 s post-exercise [51]. Hence a pronounced reduction in CMJ performance (height and power) after 180 s will reflect the diminished PCr store replenishment capacity of muscle fibers affecting the stretch-shortening cycle and force production [52]. The present observations are in good agreement with prior findings in which an effect of time in reducing CMJ height and mean power output was seen after a 30-s all-out Wingate test [28,29,30]. The decrease in CMJ performance was more pronounced at 30 s (~30%) compared to 180 s post-exercise (~10%). However, no differences between supplementation conditions were observed. 

In our study, BJ supplementation overall did not give rise to a greater fatigue index during the second half of the test or to neuromuscular fatigue as measured in CMJ tests, after the 30-s all-out sprint test. These results indicate that the improved sprint performance induced by BJ as a NO_3_^−^-rich supplement may not be accompanied by more fatigue. 

## 5. Limitations

Our study has several limitations. BJ is a NO_3_^−^-rich supplement known to increase circulating NO_2_^−^ and NO levels [7]. However, these levels were not measured before the intake by the participants of placebo or BJ. Further, the number of subjects recruited (*N* = 15), although appropriate for this type of study, limits the detection of small changes that could be the consequence of BJ administration. Finally, participants were not trained cyclists and therefore the ergogenic effects produced by BJ cannot be directly transferred to this sports modality. On the up-side, however, the inclusion of resistance trained individuals was useful to explore the physiological effects of BJ supplementation on skeletal muscle power production and to examine fatigue induced by a sprint exercise to exhaustion.

## 6. Conclusions

In conclusion, BJ supplementation produced an ergogenic effect in a 30-s all-out Wingate test in terms of increasing W_peak_, Time to W_peak_ and W_mean_, particularly during the first half of the sprint, without increasing muscular fatigue accumulation during or after this extenuating sprint exercise. These findings suggest that NO_3_^−^-rich supplements could be a suitable strategy to improve performance in sports modalities in which power and acceleration largely determine performance.

## Figures and Tables

**Table 1:** Effects of placebo or BJ intake on performance at a 30-s sprint (Wingate) test.

Variable	Placebo	BJ	*p*-Value
W_peak_ (W)	848 ± 134	881 ± 135	0.049
Time to W_peak_ (s)	8.9 ± 1.4	7.3 ± 0.9	0.003
W_mean_ (W)	641 ± 91	666 ± 100	0.023
W_min_ (W)	453 ± 64	472 ± 72	0.064
Fatigue index (FI) (%)	46 ± 8	46 ± 7	0.914

Values are means ± standard deviation. BJ, beetroot juice.

**Table 2:** Effects of placebo or BJ intake in a neuromuscular fatigue (CMJ) after a 30-s all-out Wingate test.

Variable	Placebo			BJ			Suppl.	Time	Suppl. × Time
	Pre	Post	Post-3	Pre	Post	Post-3			
CMJ_height_ (cm)	30.8 ± 4.6	19.5 ± 5.1 ^a^	25.0 ± 4.3 ^a,b^	31.5 ± 3.4	19.0 ± 4.2 ^a^	25.3 ± 4.2 ^a,b^	0.863	<0.001	0.864
CMJ_Wpeak_ (W)	50.5 ± 4.7	36.9 ± 5.9 ^a^	45.2 ± 4.6 ^a,b^	51.1 ± 3.6	36.6 ± 4.9 ^a^	44.7 ± 4.5 ^a,b^	0.947	<0.001	0.850
CMJ_Wmean_ (W)	27.3 ± 3.6	20.0 ± 4.2 ^a^	24.0 ± 3.8 ^a,b^	27.9 ± 3.3	19.6 ± 3.5 ^a^	23.8 ± 3.6 ^a,b^	0.994	<0.001	0.850

Values are means ± standard deviation. ^a^ *p* < 0.05 compared to Pre; ^b^ *p* < 0.05 compared to Post. Pre, before sprint exercise; Post, post-exercise; Post-3, 3 min post-exercise.
